# Implementing a training intervention to support caregivers after stroke: a process evaluation examining the initiation and embedding of programme change

**DOI:** 10.1186/1748-5908-8-96

**Published:** 2013-08-23

**Authors:** David James Clarke, Mary Godfrey, Rebecca Hawkins, Euan Sadler, Geoffrey Harding, Anne Forster, Christopher McKevitt, Josie Dickerson, Amanda Farrin

**Affiliations:** 1 Academic Unit of Elderly Care and Rehabilitation, Temple Bank House, Bradford Royal Infirmary, Bradford BD9 6RJ, UK; 2 Department of Primary Care and Public Health Sciences, King’s College London, 7th Floor, Capital House, Guy’s Hospital, 42 Weston Street, London SE1 3QD, UK; 3 Leeds Institute of Health Sciences, University of Leeds, 101 Clarendon Road, Leeds, West Yorkshire LS2 9LJ, UK; 4 Clinical Trials Research Unit, University of Leeds, Leeds LS2 9JT, UK; 5 Peninsula College of Medicine and Dentistry, University of Exeter, Prince of Wales Road, Exeter, Devon EX4 4SB, UK

**Keywords:** Process evaluation, Implementation theory, Stroke, Caregiver training, Normalization process theory

## Abstract

**Background:**

Medical Research Council (MRC) guidance identifies implementation as a key element of the development and evaluation process for complex healthcare interventions. Implementation is itself a complex process involving the mobilization of human, material, and organizational resources to change practice within settings that have pre-existing structures, historical patterns of relationships, and routinized ways of working. Process evaluations enable researchers and clinicians to understand how implementation proceeds and what factors impact on intended program change. A qualitative process evaluation of the pragmatic cluster randomized controlled trial; Training Caregivers after Stroke was conducted to examine how professionals were engaged in the work of delivering training; how they reached and involved caregivers for whom the intervention was most appropriate; how did those on whom training was targeted experience and respond to it. Normalization Process Theory, which focuses attention on implementing and embedding program change, was used as a sensitizing framework to examine selected findings.

**Results:**

Contextual factors including organizational history and team relationships, external policy, and service development initiatives, impinged on implementation of the caregiver training program in unintended ways that could not have been predicted through focus on mechanisms of individual and collective action at unit level. Factors that facilitated or impeded the effectiveness of the cascade training model used, whether and how stroke unit teams made sense of and engaged individually and collectively with a complex caregiver training intervention, and what impact these factors had on embedding the intervention in routine stroke unit practice were identified.

**Conclusions:**

Where implementation of complex interventions depends on multiple providers, time needs to be invested in reaching agreement on who will take responsibility for delivery of specific components and in determining how implementation and its effectiveness will be monitored. This goes beyond concern with intervention fidelity; explicit consideration also needs to be given to the implementation process in terms of how program change can be effected at organizational, practice, and service delivery levels. Normalization Process Theory’s constructs help identify vulnerable features of implementation processes in respect of the work involved in embedding complex interventions.

## Introduction

Health services research aims not only to develop effective evidence based interventions, but to understand what is required to produce replicable outcomes in the context of day-to-day practice. Addressing the problem of developing, implementing, and evaluating complex interventions within increasingly complex organizational and clinical environments, poses major theoretical, methodological, and resource challenges [1] and requires focus on several questions. Does the intervention work; is it effective? How is it intended to work; what is the theory of change underpinning it? What are the human and material resources necessary to implement and sustain it in specific health settings and how are these mobilized to deliver it? How do local, contextual features of the setting in which the intervention is introduced affect how it is implemented and shape patterns of variation in outcomes achieved. Although Medical Research Council (MRC) guidance [1] on complex interventions includes implementation as a key element of the development and evaluation process, the focus is primarily on intervention development and evaluative work.

We examine implementation as a process involving the mobilization of human, material, and organizational resources to change practice within settings that have pre-existing structures, historical patterns of relationships and routinized ways of working. This is explored through a process evaluation of the pragmatic cluster randomized controlled trial (RCT), Training Caregivers after Stroke (TRACS), which was funded separately from the trial and conducted by an independent research team. The process evaluation objectives were to: describe how the training program was implemented in different settings; provide insight into patients’, caregivers’, and multidisciplinary team (MDT) members’ experience of the training process; understand patients’ and caregivers’ perceptions of their preparedness for discharge and what shaped their views and experiences; provide data to assist in interpreting the trial outcomes; and contribute to knowledge of current clinical practice in stroke units in England. We focus on one component of the process evaluation, namely how change was introduced and implemented, which is integral to establishing 'what works’ in a complex intervention.

TRACs is one of the largest rehabilitation trials conducted to date; 930 patients and caregivers were registered. The intervention—a training program targeted at caregivers of stroke survivors, the London Stroke Carer Training Course (LSCTC), was intended to be delivered by MDT members within stroke units to secure positive outcomes for patients and their caregivers. The research questions addressed in the process evaluation included: how were professionals engaged in the work of delivering the training; how did they reach and involve those caregivers for whom the intervention was most appropriate; how was it delivered to caregivers; and how did those on whom it was targeted experience and respond to it.

This paper briefly considers implementation theories in respect of complex interventions and provides an overview of process evaluations to set the context for the study. We draw on Normalization Process Theory (NPT) [2] as a conceptual lens through which to explore those features of the implementation process that were intended to secure practice change and to engage caregivers in the program. We also consider the interaction between influential macro and micro contextual factors that affected delivery by multi-disciplinary stroke unit staff and suggest that prior focus on generative mechanisms identified within NPT can be used to inform implementation processes within complex healthcare settings.

## Background

### Process evaluations

Process evaluations are increasingly viewed as necessary elements of the methodological toolkit, alongside RCTs. The objectives of such evaluations have been variously described:

'To explain discrepancies between expected and observed outcomes, to understand how context influences outcomes, and to provide insights to aid implementation’ [1] (2008:4);

'To examine the views of participants on the intervention; study how the intervention is implemented; distinguish between the components of the intervention; monitor dose to assess the reach of the intervention; and study the way the effects vary in sub-groups’ [3] (2006:413).

Munro and Bloor [4] argue that considerable explanatory burden is being placed on this type of evaluation, which embraces enormous variation in objectives, research designs, and methods as well as depth and breadth of understanding attained. Typically, process evaluations focus on such questions as the acceptability of the intervention, whether it was implemented as intended (the fidelity 'problem’), and how and why it had its effects—and are often empirically driven. However, developing and evaluating complex interventions demands considerably more, given the multiple layers of questions posed to develop and translate evidence-based knowledge into routine clinical practice.

### Theory oriented approaches to process evaluations

Theory-oriented approaches to developing and implementing change whether at system, organizational, professional, or individual level offer fruitful conceptual tools for getting inside the 'black box’ of complex interventions; which Stame [5] describes as the 'space between the actual input and the expected output of a program’ (2004:58). These include realist evaluation [6] and 'theory of change’ approaches [7,8]. Realist evaluations explore the relationship over time between 'context’ (organizational setting and external constraints), 'mechanisms’ (how change will be achieved) and 'outcomes’ (intended and unintended consequences of change efforts). 'Theory of change’ approaches aim to explicate assumptions and causal chains linking inputs and activities to desired outcomes, comparing what is intended with what actually happens in real life. Theories of change have two components: 'programmatic theory’ based on the mechanisms that make change happen; and 'implementation theory’ that forecasts in a descriptive way the steps to be taken in program implementation. These are the focus of the process evaluation components reported in this paper.

### Implementation theories

Introducing and embedding a new technique, intervention or way of working into organizations requires understanding of what behaviours and practices need to change, what systems and mechanisms need to be put in place to support implementation and what resources are needed to achieve both. These factors, familiar to theorists interested in implementation science, may not feature prominently in the planning undertaken by trial management groups in advance of introducing interventions into clinical settings. Fixsen *et al.*[9] noted that, 'there is very little information about the processes used to gain access to and secure the cooperation of individuals, organizations, departments, and political groups. Thus, organizational and systems intervention strategies and skills represent a critical research area’ (2005:74). The 'implementation problem’ raises new challenges for the design and conduct of process evaluations [10-12]. First, implementation is more accurately conceived of as a process and not just a single event, requiring attention on what needs to occur at different stages and over time [9]. May and Finch [11] develop this further to consider the mechanisms that facilitate or hinder implementing and embedding change in routine practice. Normalization Process Theory (NPT) [2], focusing primarily on practice change, addresses explicitly the issue of how interventions are adopted, embedded, and integrated into organizational routines. Second, there is what Hawe *et al*. [13] term the 'program in context' problem: interventions or programs are introduced into organizations with varied histories, cultures, learning climates, and readiness for implementation, all of which will affect outcomes [14-16]. Pawson [17] and Campbell *et al*. [18] argue that emphasis on determining underlying causal mechanisms provides a basis for examining how organizational contexts can shape implementation of particular interventions and their outcomes. Hoddinott *et al*. [19] also suggest that contextual and organizational features of healthcare environments require more attention as these may have a significant impact on how far work groups can implement complex interventions. Additionally, individual level change may draw upon behavioral, motivational, and psychological theories to shape implementation strategies. Critical is that the theory or conceptual framework informing the development or evaluation of the intervention and implementation processes is appropriate for the level and type of change being implemented; and that change at multiple levels may draw on multiple theories [20].

### TRACs trial: program theory

Increasingly, there is an expectation that family members will provide support for stroke patients following their return home [21-24]. Despite improvements in outcomes, many patients have some residual disability and require assistance with daily living activities, including washing and dressing, eating, drinking, and walking [23]. Informal caregivers face the dual challenge of coming to terms with the sudden onset and subsequent disabling impact of stroke and the realization that the person may require long-term support [25]. There is considerable evidence to suggest that caregiving insofar as it results in role captivity, role overload, and loss of reciprocal companionship, leads to considerable stress and sense of burden [26,27]. Interventions that reduce caregiver burden and stress are therefore a high priority for stroke service providers [28].

### The 'theory of change’ underpinning caregiver training

Information and skills training to provide physical care for stroke patients is among caregivers’ most important pre-discharge needs [26]. Preparation and support has been deficient in meeting these needs; and caregivers’ ability to provide such care is not normally assessed by health professionals [26,27,29-31]. Moreover, evaluations of interventions providing psychological, emotional, and informational support report little impact on patients and only modest improvements for caregivers [31]. The empirically based LSCTC was designed to train caregivers in practical skills for supporting patients. When tested in a single center the LSCTC was effective in decreasing burden, anxiety, and depression for the caregiver, in improving psychological outcomes for patients, and in reducing overall costs [28].

We found no evidence that the Kalra *et al*. [28] or the TRACS trial team theorized the ways in which the intervention was expected to 'work’ in practice. However, the implicit 'theory of change’ on which the LSCTC seems to be premised can be expressed as follows: stroke brings about physical and psychological impairments that are beyond the experience and skills of most caregivers; a knowledge and skills gap will be present. Teaching basic skills in moving and handling, how to support activities of daily living, developing understanding of the causes, and psychological and physical consequences of stroke will enable caregivers to support stroke survivors in the home. This should result in reductions in burden, stress, anxiety and depression, in improvements in social engagement, and in overall activity. In the Kalra *et al*. [28] study, knowledge of the ineffectiveness of existing interventions appears to have led to the development of the competency-based training program (the LSCTC) delivered within the stroke unit prior to discharge. In this model, it was expected that caregiver training will contribute to the work of rehabilitation. In the single-center study, the LSCTC was designed, managed, and delivered by a small, committed, and enthusiastic team (an occupational therapist, physiotherapist and (nurse) stroke coordinator).

### TRACS- a modified training program

The TRACS trial tested whether a modified version of the LSCTC introduced into usual practice in 18 stroke units could replicate observed improvements in clinical outcomes and cost effectiveness with a more diverse patient and caregiver population (18 control units provided usual care based on national clinical guidelines [32]). Process Evaluation Sites were independently selected by Clinical Trials Unit staff using a purposive sampling frame of control/intervention (four intervention, four control), quality of care (based on 2006 Stroke Sentinel Audit scores), and geographical site (four English regions) (Table 1). The modified LSCTC was a 14-item competency based program, intended to be delivered by MDT in intervention units. Six of the 14 items were mandatory, *e*.*g*., providing information on the nature of stroke and the importance of medication adherence. Remaining items were addressed based on individual caregiver need, *e*.*g*., providing nutritional support for patients with swallowing difficulties. Caregiver competence was to be assessed by team member(s) who had provided training using a standardised training record. A follow-up visit or phone call to help caregivers adapt knowledge and skills taught, to the home, completed the intervention (Table 2).

**Table 1:** Stroke units participating in the TRACS trial and process evaluation study

**Region**	**Trial arm**	**Hospital type**	**Unit type**	**Total bed no.in 2007**	**Stroke rehabilitation bed no.in 2007**	**Rehabilitation bed no 2010**	**Therapy treatment room location**	**Early supported discharge scheme**
North West	Control	Acute	Combined	20	12	12	On unit	N
Peninsula	Control	Community	Rehabilitation	24	24	19	On unit	N
South East	Control	Acute	Combined	20	12	12	Off unit	Y
Yorkshire	Control	Acute	Combined	28	16	16	On unit	Y
North West	Intervention	Acute	Rehabilitation	18	18	18	On unit	N
Peninsula	Intervention	Community	Rehabilitation	23	11	23	On unit	
South East	Intervention	Acute	Combined	26	23	23	Off unit	Y
South East	Intervention	Acute	Combined	24	16	16	On unit	N
Yorkshire	Intervention	Acute	Combined	20	20	20	On unit	N
Yorkshire	Intervention	Acute	Rehabilitation	24	24	24	Off unit	N

**Table 2:** LSCTC training components

**The caregiver has demonstrated a knowledge and understanding of:**	
1)	His/her relative having had a stroke (mandatory)
2)	What a stroke is (mandatory)
3)	His/her relative’s specific stroke related problems. Possible incomplete recovery and residual unresolved problems:
a)	Communication and reading
b)	Cognition
c)	Personality and mood changes
d)	Diet and swallowing
e)	Vision
f)	Personal Activities of Daily Living (PADL)
g)	Transfers and Mobility (as appropriate)
4)	The importance of a healthy lifestyle and secondary preventions:
a)	Control of blood pressure
b)	Use of Aspirin / Warfarin or similar
c)	Smoking
d)	Appropriate diet, including prevention of excess weight gain
e)	Exercise
f)	Pain Management (mandatory)
5)	Dietary needs and feeding techniques:
a)	Special diet
b)	Techniques to assist eating, including use of specialist equipment if necessary (as appropriate)
6)	How to communicate with dysphasic relative (as appropriate)
7)	How to manage relative’s personal washing, dressing, toiletry needs (as appropriate)
8)	The importance of limb positioning and the management of pressure areas and skin integrity (as appropriate)
9)	Continence management (as appropriate)
10)	Bowel management, fluid and dietary intake for the prevention of constipation (as appropriate)
11)	Appropriate techniques and ability in:
a)	Safe transfers
b)	Safely assisting mobility
c)	Floor routine following a fall
d)	Safely assisting in climbing stairs
e)	Good use of a wheelchair
f)	Use of aids (as appropriate)
12)	The importance of compliance with medication (including supervision of self- or routine medication) (mandatory)
13)	Post discharge arrangements and where and whom to seek help from after discharge (mandatory)
14)	Adapting the knowledge and skills taught to the home environment following discharge (follow-up visit or phone call) (mandatory)

MRC guidance [1] on developing and evaluating complex interventions, notes that these 'are usually described as interventions that contain several interacting components’ (2008, p. 6). The original and modified LSCTC epitomise a complex intervention. This is apparent in the heterogeneity of patient and caregiver needs and circumstances and the tailoring of the intervention in response to these; in the requirement for 'buy-in’ at different professional and organizational levels; in the collective and individual behaviors of MDT members delivering training; and in the willingness and capacity of caregivers to engage with stroke teams and participate in training. Complexity is also evident in the multiple outcomes anticipated at different levels; these would be produced through long implementation chains, operating through recursive, feedback loops, involving a non-linear and multi-directional process of change [33,34].

### TRACs trial results

The TRACS trial commenced in February 2008 and ceased recruitment in February 2010. The trial results are reported in full elsewhere [35]; a brief summary of the main findings is reported here to place our process evaluation findings in context. Results at six months demonstrated no clinical or statistical differences between groups on the primary outcomes of functional independence (patients), or caregiver burden. Similarly, for the range of secondary outcomes measured at 6 and 12 months, no clinical or statistical differences were evident. The single-center trial outcomes [28] were not replicated in TRACS. To assist in understanding the trial outcomes, we report selected findings from the process evaluation. Specifically we examine how the intervention was implemented to effect practice change within stroke unit environments, how practitioners were engaged in the work of delivering the LSCTC, and how they involved caregivers in the program.

## Methods

The process evaluation began in February 2009. Ethical approval was gained for all sites (National Health Service Research Ethics Reference: 08/H1307/104). Participants provided written informed consent prior to observations and interviews; process consent was sought prior to and during observations.

The study was conducted initially in a sub-sample of eight stroke units in four English regions selected from the 36 sites participating the TRACS trial. Sites were independently selected by Clinical Trials Unit staff using the same selection process as in the trial (Table 1). Participant eligibility were as used in the TRACS trial [35].

To address the process evaluation objectives, we employed an ethnographic approach, combining observations, interviews with MDT members, patients, and caregivers, and documentary analysis. In intervention units, we evaluated how the LSCTC was understood by staff and received by caregivers; what factors facilitated or inhibited its implementation; and caregivers perceptions of the impact of training on them. In control units, researchers recorded usual practice including any instances of caregiver training. Four researchers each undertook fieldwork over six months in two Stroke Units (SUs); to test emerging interpretations of data, observations were later conducted in two additional intervention units over a further three months.

We utilized an observational framework that sought qualitative data entries on features of clinical practice determined prior to study commencement. The framework encouraged observations of unplanned and emergent areas of practice related to the implementation of the LSCTC and progressive focusing on specific aspects of caregiver training, *e*.*g*., instances of caregiver practice under supervision. Observational records were supplemented by field notes, including researchers’ reflexive accounts [36,37] of how conducting observations impacted on participants in specific instances and over time. Over 1,200 hours of observations were completed. Observation included therapy, nursing and medical practice, team and family meetings, home visits, some outreach therapy, informal interactions between staff, between staff and patients, and between patients and family members. We observed patients with different kinds and levels of impairment. Observations took place on weekdays because staff indicated caregiver training did not routinely occur at weekends. Observations occurred at different times of the day, normally in three to four hour blocks between 0700 and 2200. Patient/caregiver dyads to be interviewed post-discharge were a specific focus of observations.

Semi-structured interviews were conducted with MDT members after TRACS trial recruitment ceased in February 2010 (n = 53). A purposive sampling approach was used to recruit staff at different grades, with different levels of experience, staff who were present throughout the TRACS trial and some who were on training rotations and less experienced with the LSCTC. Staff beliefs about caregiver training, their understanding of the LSCTC, and their preparation for and engagement with the intervention were explored. Interviews were also undertaken with a sub-sample of patient/caregiver dyads three months after discharge from hospital (n = 37 pairs). Potential interviewees were identified by researchers during the observational phase, and written informed consent was sought to interview them post hospital-discharge. A record of socio-demographic characteristics and impairment (disability, cognition) status was made. When sufficient patients were available, purposive sampling was used to ensure that a diverse sample was recruited (Table 3). Patients’ and caregivers’ in hospital and overall post discharge experiences were explored before focusing more specifically on their experiences of caregiver training and whether this prepared caregiver and patient for life at home. All interviews were audio-recorded with participants’ permission and subsequently transcribed.

**Table 3:** Participant characteristics

**Patients**	**Intervention(n=16)**	**Control(n=22)**
**Age(yrs)**		
Mean (s.d.)	69(15)	74 (15)
Median (Range)	73 (38,87)	74 (21,99)
**Sex**		
Female (%)	6 (38)	14 (64)
Male (%)	10 (62)	8 (36)
**Language ability %**		
Normal	9 (56)	12 (54)
Aphasia	5 (31)	7 (32)
Dysarthria	2 (13)	3 (14)
**Barthel score @ Discharge**		
Mean (sd)	12.3 (5)	9.8 (5)
Median (range)	12 (1,20)	8.5 (1,20)
**Caregivers**	**Intervention(n=16)**	**Control(n=21)**
**Age (yrs)**		
Mean (s.d.)	59.9 (13)	67 (13.9)
Median (Range	61 (42,82)	70 (33,90)
**Sex**		
Female (%)	11 (69)	11 (52)
Male (%)	5 (31)	10 (48)
**Staff**	**Intervention(n=24)**	**Control(n=30)**
OT	6 (25)	4 (13)
Physiotherapist	5 (21)	6 (20)
SALT	2 (8)	1 (3)
Nurse	6 (25)	9 (30)
HCA	1 (4)	1 (3)
Medical Staff	0	1 (3)
Social Worker	0	1 (3)
Stroke Association Worker	2 (8)	2 (7)
Dietician	1 (4)	0
Unknown	1 (4)	5 (17)

Documentary analysis was undertaken using therapy, nursing, or MDT records for these dyads to identify recorded caregiver training. After on-site data collection ceased, we also accessed intervention compliance data for each unit through training record returns.

The process evaluation generated a very large volume of data. To manage and effectively explore this rich data set, we drew on grounded theory analytic methods across all data sets; this included constant comparison, progressive focusing of field observations, and memo-writing to construct categories and sub-categories [38,39]. Researchers met every eight weeks to review analytic categories and develop interpretations that might provide a credible and plausible account of patterns in the data. Emerging interpretations were also reviewed by steering group members every twelve weeks. We employed NPT [2] concepts as a sensitizing framework to develop insight into those factors that facilitated or presented barriers to introducing and embedding the practice change in stroke units. Use of sensitizing concepts are not antithetic to a grounded theory approach; rather they suggest lines of enquiry to pursue that do not act as a straight jacket thereby closing off fruitful areas to explore that might not have been anticipated [38,39]. Of the different theories of implementation, NPT was selected for its focus on individual and collective practices within organizational settings.

### NPT as a sensitizing framework

Individual and collective components of complex interventions are important areas for research, specifically how those charged with responsibility for implementation understand and deliver them. Even where interventions are considered as building upon or enhancing existing practice, planning for and introducing complex interventions into healthcare settings represent a program change that will necessarily have implications for organizations, their staff, and service users [9,15,19]. These issues are highlighted in NPT [11] as central to understanding implementation and how integration of innovations proceeds. May *et al*. [2] argue two 'problems’ have to be considered by process evaluations, the 'process problem,’ seen as part of implementing change in professionals’ thinking and behavior, and the 'structural problem’ that is concerned with integration of a new practice into existing settings [2]. NPT identifies four generative mechanisms that explicate how interventions are embedded and 'normalized’ within routine care. These are: coherence, cognitive participation, collective action, and reflexive monitoring; that are singly and together envisaged as interacting with the healthcare context [2] (See Table 4 for more detail on these mechanisms). We reviewed the process evaluation data through the lens of these generative mechanisms, considering them in terms of MDT members’ actions and also those of patient/caregiver dyads; we draw from the extensive observational data generated. Interview data are reported elsewhere.

**Table 4:** **Normalization process theory**: **the work of implementation**

**Four interrelated generative mechanisms (after May &Finch [**[11]**]; May*et al*. [**[2]**]).**		
**Contexts**	**Generative mechanisms**	**Explanation**
The generative mechanisms are considered to be in dynamic interaction and are influenced by individual and wider, professional, local practice and organizational contexts	**Coherence**	Coherence [individually and collectively]relates to: how the work that defines and organizes a practice/intervention is understood, rendered meaningful and invested in, in respect of the knowledge, skills, behaviours, actors and actions required to implement it.
	**Cognitive participation**	Cognitive participation relates to: commitment to and engagement of participants with the intervention. Do participants view the intervention as something worthwhile and appropriate to commit their individual time and effort [signing up] to bring about the intended outcome?
	**Collective action**	Collective action relates to: the work that will be required of participants to implement the intervention, including preparation and/or training. How far will existing work practices and the division of labour have to be changed or adapted to implement the intervention? Is the intervention consistent with the existing norms and goals of the groups, the workplace and overall organization [this is policy, practice and service user linked]
	**Reflexive monitoring**	Reflexive monitoring relates to: participants’ individual and collective on-going formal and informal appraisal of the intervention and its benefits for participants, in relation to realizing individual and organizational goals.

## Results

### Training model

To prepare teams to deliver the LSCTC in 18 intervention units across four regions two full-day workshops were held (one month apart) for two or three representatives from each unit. These MDT members volunteered to undertake initial training and then cascade training to MDT members in their own units. All intervention units were represented at day one and day two, except for one unit that had no staff member at day two. Workshops were led by three professionals who implemented the original LSCTC. A participative format, getting representatives to focus on examples of competencies and how these might be taught and assessed on their units was adopted. Between workshops, attendees were to begin cascade training, but not expected to implement the LSCTC program immediately; this was to occur gradually over three to four months before TRACS trial recruitment commenced.

Critical to implementation was how other stroke unit staff were engaged with the LSCTC through cascade training, this relates to what the NPT model terms coherence or making sense of the intervention before it is implemented; and how far existing working practices within stroke units required adaptation to bring about the cognitive participation and collective action that would be required to successfully embed the LSCTC in practice.

### Cascade training

Cascade training is commonly used in the National Health Service (NHS), with those receiving primary training referred to as change champions; their selection is considered critical to the method’s effectiveness [40]. The majority of MDT members attending workshop training in the TRACS trial were senior, experienced therapists and nurses with the necessary skills to deliver caregiver training, and authority in respect of MDTs in their units (Table 5). They had direct access to original LSCTC staff; they had a focused and extended period of time to think through and engage with program elements and to discuss implementation in their units. They had time to make sense of the LSCTC, to develop coherence in a group context, and to develop individual and small group commitment to it; progress to cognitive participation, *i*.*e*., determining whether implementing the LSCTC was worthwhile. This required consideration of how far existing practice could accommodate the intervention or how far practice might require adjustment and how the effectiveness of the proposed change would be monitored. These represent the work of collective action and reflexive monitoring, which are considered to be integral to embedding practice change [2]. In contrast, MDT members participating in secondary cascade training received much shorter introductions to the LSCTC. While the average length of secondary sessions was 40 minutes, some were as short as 10 or 15 minutes, albeit these were repeated in each unit. Opportunities to make sense of, develop commitment to, and ownership of the intervention through cascade training for MDT members at unit level were limited and were further constrained by the division of labor within stroke units.

**Table 5:** **TRACS**: **attendance at pre**-**trial LSCTC training workshop 1** &**2**

**DAY 1**							
**Band**	**PT**	**OT**	**Nurse**	**SALT**	**Dietician**	**Stroke Co**-**ordinator**	**Stroke physician**
5		1	7				
6	9	8	2				
7	8	3	7	2	1	1	
8	2		1				
Unknown	1	1	1				1
**Total**	**20**	**13**	**18**	**2**	**1**	**1**	**1**
**DAY 2**							
**Band**	**PT**	**OT**	**Nurse**	**SALT**	**Dietician**	**Stroke Co**-**ordinator**	**Stroke physician**
5			4				
6	4	6	2	1			
7	2	3	4				
8	1						
Unknown		1					
**Total**	7	10	10	1	0	0	0

All units were represented at day 1 and day 2 except one unit that had no staff member at day 2.

### The division of labor in stroke units

The cascade-training model was not universally effective in securing coherence and cognitive participation for all MDT members in the intervention units studied. The division of labor in most intervention units impacted negatively on cascade training and LSCTC implementation. Physiotherapists (PTs), occupational therapists (OTs) or the trial manager provided most cascade training; nurses led training in only three of 18 units. Across units there was variable attendance, with lower rates of attendance by nurses, physicians, social workers, and psychologists. Therapists provided the bulk of cascade training; this may have implied ownership of the intervention by them. Organizational factors also impacted on attendance. Therapy work is typically organized around activity with patients at specified times. Therapy sessions could be delayed or postponed, enabling therapists to provide or participate in LSCTC training. This contrasted with nurses’ working practices; they reported that attending training during shifts was difficult due to continuous demand for nursing care. Routine MDT working practices could have provided additional opportunities for MDT members to make sense of, assess the relevance of, and engage with the intervention. We observed this occurring for rotational and newly appointed therapists as they were introduced to the LSCTC and caregiver training. However, for other MDT members including nurses, unidisciplinary working practices and the particular locations in which therapists engaged with caregivers (off unit or in therapy rooms), meant that non-therapy MDT members did not observe and did not participate in caregiver training. A DVD of the training workshops and a training manual was located in each intervention unit; observations recorded no instances of these being used to support cascade training and interviewees made no reference to these resources.

### Implementing the LSCTC

The LSCTC was presented as an intervention reliant on collaborative inputs from all MDT members. LSCTC competencies can be interpreted as reflecting the existing division of labor in stroke units. The focus of specific competencies, *e*.*g*., on transfers and mobility or compliance with medication was associated by MDT members with particular disciplines; the first example was with PTs and OTs, the second with nurses. Dividing responsibility for delivering different competencies between the team was perhaps logical and consistent with the division of labor observed. However, this relied heavily on individual professionals, their disciplinary work groups, and the MDT developing ownership and taking responsibility for delivering, recording, and reporting caregiver training. All intervention units displayed characteristics of established MDT working, including weekly structured meetings to agree goals for patients and co-ordinate MDT member inputs [41]. However, as reported in other studies, outside of those events, MDT members in almost all units tended to work in parallel unidisciplinary groups [42,43]. Parallel working in itself was not problematic provided that the required caregiver competencies were addressed by a team member. However, although observations identified that team members regularly reported to each other on their actions against patient goals, this largely did not include discussion of caregivers’ achievement of LSCTC competencies. No mechanism appeared to exist for review of LSCTC delivery in any of the intervention units. While this was not required by the trial, its absence led to assumptions about what training had or had not been provided, which was reflected in incomplete implementation of the LSCTC.

### Cognitive participation, collective action and reflexive monitoring

A critical factor in implementing complex interventions is the degree to which professionals perceive that the intervention will be consistent with existing work, will necessitate practice change, or will require additional skills and time [44]. In short, professionals may question whether the work of implementation will be worthwhile if they cannot see that the benefits of the change outweigh the time and skills costs involved in changing practices. In keeping with the pragmatic nature of the TRACS trial, workshops had emphasized that the LSCTC, including training and assessment of caregiver competence, could be incorporated into routine practice. The only identified changes to existing work were the specification of six mandatory training competencies and documentation of training and assessment of caregiver competence in a record designed for the trial. This approach was premised on an assumption that stroke unit MDTs were already engaged in structured caregiver training to some degree. This assumption, while consistent with the perceptions of many MDT members, was not supported by observations in intervention and control units. In reality, LSCTC implementation required some changes to existing practice (*e*.*g*., assessing caregiver competence) for all intervention units and had additional time implications for most MDT members.

Observational and interview data indicated that soon after cascade training, variations were evident in respect of coherence, cognitive participation, and collective action between disciplines and across units. Senior therapists in most units were more likely to understand the requirements of the intervention; and in informal conversations they noted that for less experienced therapists, working through relevant LSCTC competencies and using the training record as an aide memoire, enhanced provision of training for individual caregivers. In contrast, senior nurses commonly expressed the view that the LSCTC intervention was consistent with good practice, was an important team goal, and that competencies associated with nursing (*e*.*g*., training in medication use or nutritional support) were already being delivered. In practice, neither observations nor LSCTC training records demonstrated that nurses were consistently engaged in implementing the intervention. Most junior nurses, healthcare assistants (HCAs), some junior therapists, and most junior doctors showed little or no knowledge of the intervention. In units where competency records were infrequently completed, lost, or not completed at all, therapists and senior nurses reported that caregiver training was still occurring; these claims were often not supported by our observations.

### Documenting caregiver competency

Therapy and nursing inputs were typically recorded in separate disciplinary records as well as in medical notes; and in some units, also in shared MDT notes. In units using electronic patient records (EPR), with shared access, separate sections were maintained by each discipline and rarely reviewed by other disciplines. Recording training and competency assessment in a separate (trial) record was commonly referred to as problematic. In units piloting the Lorenzo NHS EPR, the TRACS caregiver training record was one of several documents recording the same aspect of MDT members’ work. The training record was different from other records because it documented work with caregivers rather than patients. How staff managed this varied considerably. At one end of the spectrum, the TRACS record was completed by senior therapists who kept records in the therapy area and routinely checked that other team members had taught and assessed caregiver competencies. In other units, TRACS records were stored at a shared workstation, and a therapist, senior nurse, or local research practitioner reminded colleagues to complete the record. In one unit, staff reported that failure of one group to complete their section of the record resulted in remaining MDT members deciding to stop record keeping. Inconsistency in completing documentation is not necessarily evidence of lack of engagement of MDT members with the LSCTC; but it poses important questions about accomplishments in respect of cognitive participation, collective action, and reflexive monitoring at team level in some units. These issues are indicative of the 'process problem,’ some of which were features of participants’ interaction with the trial teams’ implementation strategy; others related to the organizational and professional contexts into which the intervention was introduced. We now consider contextual features influencing stroke units during implementation of the intervention.

### Contextual factors impacting on implementation of the LSCTC

Shiell *et al*. [45] draw a distinction between complex interventions that involve a range of identifiable but interacting components, the effect of which can be difficult to separate out, and complex systems such as hospitals or primary care settings. In these settings, complexity is a property of the interaction between local contexts, the behavior and actions of staff, and service users in particular organizations and local structures. Such systems are never static but are subject to variation, change, adaptation, and development over time as a result of external and internal factors [14]. There is international consensus on the essential characteristics of stroke units [41], but there is less certainty about their specific contribution to the improved outcomes associated with organized stroke care. Stroke units in the TRACS trial met five key stroke sentinel audit criteria [46] (Table 6).

**Table 6:** **Stroke unit key criteria (RCP national sentinel audit for stroke 2006 **[46]**)**

1)	Consultant physician with responsibility for stroke
2)	Formal links with patient and carer organizations.
3)	Multidisciplinary team meetings at least weekly to plan patient care.
4)	Provision of information to patients about stroke.
5)	Continuing education programmes for staff.

Despite the presence of these characteristics, variability is likely due to differences in staff expertise and skill mix, local management, and organizational policies and infrastructure [47-49]. Such variation was evident in the units observed. Major changes have occurred in the organization and delivery of stroke services in the last five years [41]. Service quality improvement initiatives have been driven by the Stroke Improvement Program [50] and the National Sentinel Audit for Stroke [41], which reports on the quality of stroke care achieved against agreed criteria for stroke services [46].

Recruitment to the TRACS trial began in February 2008, soon after the National Stroke Strategy (NSS) [51] provided impetus and funding to improve stroke services in England. Also, Stroke Research Networks designed to increase participation in stroke research were supporting an expanding portfolio of RCTs in stroke units across England. In some intervention units, LSCTC implementation competed with other stroke research or service improvement projects for MDT members’, patients’, and caregivers’ commitment and participation. Change, development, and quality improvement are inherent features of modern health services but we contend that stroke units were subject to numerous local and national pressures that impacted on LSCTC implementation. Some pressures were similar for all units; for example, all hospitals were working toward increasing the number of stroke survivors spending part or all of their inpatient stays on a stroke unit, and most were planning or introducing thrombolysis services [41]. Other pressures reflected local priorities, for example the introduction of early supported discharge (ESD) schemes or service reconfigurations requiring changes in staff location or roles.

Kaplan *et al*. [14] define context as anything not part of the intervention or the implementation process and that may include factors relating to the characteristics of the organizational setting, the individual, his or her role in the organization, and the wider environment. Thus, contextual factors may operate at a micro-level as for example in relation to groups of people that work together routinely to provide care as in a stroke unit. Such units moreover are embedded within miso-level organizations as hospitals that in turn are located within larger or macro-level environments that impinge on them. It is the combined and interactive effect of particular contextual factors at these different levels that will affect variation in implementation. Here, we focus on two examples of contextual factors at SU level where the impact of national and local policy decisions, staff responses to them, and their effect on LSCTC implementation, differed markedly.

All intervention units were examining ways to respond to NSS [51] recommendations outlined above. In one unit providing acute and rehabilitation care, direct admission of patients to the stroke unit were prioritized. This MDT was working with more acutely ill people, and there was pressure to transfer non-acute patients to a generic rehabilitation unit, sometimes more quickly than staff considered appropriate. For therapists, the net effect was that they had insufficient time to assess the needs of some patients and reduced time to work with them due to the emphasis on early transfer or discharge of non-acute patients. Caregivers for this group proved unlikely to participate in the LSCTC. Observations indicated that these issues were compounded by extended staff sickness, experienced senior staff leaving, and delays in their replacement. These factors directly affected leadership, management, and staff morale, and meant LSCTC implementation was only one of many demands faced by the MDT. Communication and interprofessional relations between MDT members were observed as strained due to the increasingly stressful workload and the staffing pressures highlighted. Informal conversations indicated that historical problems related to utilization of healthcare and rehabilitation assistants by the team were an additional source of strain.

While nurses and therapists in this unit were affected similarly by the above pressures, they expressed different views about LSCTC implementation. This reflected, in our view, differences in respect of coherence and cognitive participation. Most therapists concentrated their efforts on assessment and establishing short-term goals. Some senior therapists argued that despite non-completion of training records, caregiver training was being provided where opportunities existed. Others questioned the feasibility of training caregivers in what were regarded as complex skills, arguing that much more time would be necessary to develop confidence and competence in some of them. The majority of nurses and HCAs appeared to be unaware of the LSCTC and did not routinely deliver caregiver training, prioritizing instead physical care activity. Senior nurses were aware of the LSCTC; however, the ward sister expressed concern about the capacity and readiness of nurses and HCAs to participate in caregiver training. Lack of knowledge of the interventions was not confined to nurses and HCAs; therapists on training rotations and a senior therapist all claimed they were unaware of the trial. Working relations between team members in the unit were already strained so when local pressures changed admission practices and staff shortages occurred, implementation of the LSCTC did not progress. Organizational and professional conditions at SU level for embedding the LSCTC were unfavorable, coherence was not evident, and progression through cognitive participation was not apparent at individual or team level.

In another hospital subject to the same external pressures, there were separate acute and rehabilitation units; therapists and physicians worked across both. Local policy prioritized development of an ESD scheme provided by existing staff. This placed considerable demands on senior therapists who became the main providers of the ESD in patients’ homes. With no replacement staff to cover inpatient work and with ESD visits taking therapists out of the unit for up to two hours, these MDT members could have withdrawn from LSCTC implementation in order to manage increased workload demands. In practice, they appeared to interpret ESD as providing opportunities to train and assess caregivers’ competence in the home. For these MDT members, cognitive participation, collective action, and reflexive monitoring were evident. The LSCTC, despite requiring additional work to deliver and document, was perceived to be consistent with service goals, with local policy intentions and NSS [51] standards. Despite increases in workload, this setting experienced stability and continuity in leadership and LSCTC champions engaged with caregiver training. Hoddinott *et al*. [19] termed this kind of response a 'can-do culture’; there was evidence that in this and other units, responses to change were characterized by this kind of problem-solving approach. Organizational culture in this unit facilitated implementation of the LSCTC particularly by therapists. However, collective action and reflexive monitoring were confined to the therapy team, nurses’ observed lack of engagement with the LSCTC was not altered by the introduction of the ESD scheme.

These contextual factors were partly consequent on managerial and resource allocation decisions; nonetheless, they impacted directly on MDTs, on implementation of program change through the LSCTC, and indirectly on patients and caregivers. Such factors were present to a greater or lesser degree across all intervention and control units. However, stable and established MDTs generally were able to 'make the change’ [9,19] as well as maintain implementation of the LSCTC. MDTs in the units observed existed on a continuum from strong collaborative cultures to loose confederations of different disciplines; existing research confirms these findings [42,52,53]. MDT working is considered to be a key contributor to improved outcomes after stroke but this kind of working can be problematic for interventions that are reliant on collective action if no explicit agreement on activity allocation is made, or if assumptions are made about completed activity without verification.

## Discussion

This study is one of the largest process evaluations to be undertaken in stroke rehabilitation. Sustained use of observations in ten stroke units enabled close examination of the actions and interactions taking place in post-stroke rehabilitation, including caregiver involvement. We sought not to participate in that practice, but to interact with MDT members, patients, and caregivers as they engaged in the work of recovery and rehabilitation. Supplemented by interview and textual data, observations provided insight into the reality of day-to-day MDT activity as that was experienced by staff, patients, and caregivers. Sustained observations may raise concerns that the presence of observers can impact on and influence those observed and so confound trial results. In this study, the presence of observers did add to the workload of MDT members, in that they explained and accommodated our presence as well as explained their practice as it occurred. However, we did not gather any evidence that indicated that the presence of observers resulted in increased compliance with the LSCTC. This could have occurred through MDT members being more aware of the intervention. There was no observed change in behavior related to caregivers. This was confirmed through review of compliance data for the TRACS training records that indicated no increase or decrease in intervention unit caregiver training activity during the observational period. Due to the delay in commencing the process evaluation study, observers were not present at initial training or initial implementation of the LSCTC. However, observations and informal and formal interview data provided strong supporting evidence consistent with MDT members claims regarding the ways in which the LSCTC intervention was introduced and how staff engaged or did not engage with caregiver training during the course of the trail.

Features of LSCTC implementation across the sample of trial sites observed, posed questions as to the implementation factors that point to vulnerability of the intervention. First, it was evident that in the majority of intervention units, achieving coherence in respect of the LSCTC was not perceived (in advance of implementation) as difficult because it was assumed that MDT members were already engaged to some extent in promoting caregiver involvement in supporting patients in hospital. Moreover, by combining education and sensitizing senior staff to review taken for granted practices, the initial training program engaged and motivated them to participate in a change process and facilitated coherence at their level. Extending this knowledge and understanding to the wider MDT and securing individual and collective sign-up to the LSCTC was more problematic; in the majority of units, coherence and progression to cognitive participation did not move beyond senior therapists and some senior nurses. That participation in training sessions was more variable among nursing and HCA staff may have reflected the fact that nursing work has a structured rhythm organized around the provision of time-sequenced care, treatment, and observational tasks that are also prone to disruption as a result of crisis events. To engage such staff in cascade training would require an approach that takes account of their work rhythm. Because cascade training was most often delivered by therapists with nurses claiming difficulty in being able to attend training, nurses’ participation in the training for delivery of the LSCTC was not highlighted, reinforcing the conception of caregiver training as primarily therapy-related work.

Second, organizational legitimacy in the sense of staff viewing caregiver training as a problem that required specific action was not evidenced across disciplinary groups. Nursing staff, in particular, with whom patients and caregivers had most sustained day-to-day involvement, generally considered that the LSCTC was no more than what they did routinely whereas our evidence contradicts this perception. Establishing a process whereby staff can compare their existing practice with caregivers, and assess the nature of the changes required to implement a new model such as the LSCTC intervention, for example through peer observation and review, is likely to be essential for cognitive engagement and as a precursor to collective action. Further, development of systems and mechanisms to establish the LSCTC within routine care would be necessary to translate cognitive engagement into collective action. For example, systems for identifying caregivers for training, the point in the in-patient episode that it should commence, and mechanisms for co-ordinating team activity with caregivers linked with patient goals and discharge planning. Elements of these occurred spasmodically in the main, and were primarily limited to particular disciplinary groups (mostly therapy). Only in one unit, a dedicated rehabilitation unit where interdisciplinary working practices and nursing and physician engagement were reported by staff, did they appear to occur consistently and systematically across the whole MDT. Variation in the extent to which these systems and mechanisms were in place within and between disciplinary groups, helps to explain some of the variation in implementation between sites. Finally, because achievement of caregiver competencies were not linked with goal setting and discharge planning more broadly, evidence of benefits given the resources expended (reflexive monitoring), were not clearly visible to staff. Feedback on the impact of caregiver training in the home setting, where supporting the patient was part of caregivers’ everyday activity was not available to the majority of MDT members.

This paper has addressed only some features of the implementation process relating to the TRACS trial, namely how practitioners were engaged in the program of change in stroke units. It has not, for example, touched on how caregivers were targeted and work with them pursued. Even so, in its focus on individual and collective work that needs to be accomplished to implement change in routine practice, NPT used as a sensitizing framework has value. There are commonalities and overlaps in the mechanisms in NPT that are hypothesized to affect implementation and embedding in routine practice and those identified in other models that have been derived from research on service improvement initiatives in healthcare [54,55]. We found NPT constructs were helpful in identifying problematic or vulnerable features of implementation processes in respect of the work involved in embedding a complex intervention such as the LSCTC. Even so, our evaluation also highlights other features of implementation that are less successfully captured through NPT. While May *et al*. [2] acknowledge that the NPT generative mechanisms are in dynamic interaction with local contexts and external drivers, the framework primarily addresses the mechanisms. Indeed, the theory tends to place undue emphasis on individual and collective agency without explicitly locating this within, and as shaped by, the organizational and relational context in which implementation occurs.

The trial sought to be explicit about the nature of the settings within which the intervention was implemented, drawing on Stroke Sentinel audit criteria to establish that the units within the study all met the minimum criteria of what was a stroke unit. What is revealed through the process evaluation is the speed and impact of national policy and organizational changes at national and local level that substantially altered the nature of the care environments within which the intervention was delivered with impact on implementation (e.g. shift of rehabilitation to other settings). With regard to the LSCTC, local contextual factors, including organizational history and relationships in interaction with external policy and service development initiatives, impinged on implementation in unintended ways that could not have been predicted through focus on mechanisms at the level of individual and collective action at unit level. The LSCTC was predicated on the assumption that the work of training caregivers was appropriately located within specialist stroke units that provided rehabilitation to facilitate the transition from acute care to the person’s own home. Pressure on acute services on the one hand and the emergence of a range of alternative settings within which rehabilitation was provided on the other, highlighted that continuity of LSCTC engagement with caregivers was necessary to secure competencies through co-ordination with those delivering rehabilitation in whatever setting this was provided.

In some sites, continuity was creatively achieved through ESD schemes that involved in-hospital therapists continuing to work with patients at home. More typically, discharge of stroke survivors to general rehabilitation or to intermediate care marked a premature end (or failure to commence) the intervention. Hoddinott *et al*. [19] note that the reasons why complex interventions become embedded and work in some places and not others are diverse and complex, but commonly involve barriers and facilitators at the human resource and team level. In stable organizations with co-located, committed, enthusiastic, and skilled teams, change is an accepted part of service provision, and evidence-based interventions are more likely to become embedded. The interplay between these contextual elements is significant in understanding how complex interventions are introduced and engaged with by staff in healthcare settings [19,56]. Further, the NPT framework does not appear to place sufficient emphasis on those who receive complex interventions, especially when in contemporary healthcare settings the 'service user’ is referred to as a partner in care. The conception of the LSCTC as facilitating a partnership between caregivers and staff in supporting the stroke survivor added another layer of complexity to implementation as well as posing different challenges with regard to the operation of the NPT generative mechanisms.

## Conclusion

We have highlighted the complex interrelationship between intervention components, implementation strategies, and participants who delivered or received the LSCTC intervention. The process evaluation led us to draw attention to the following factors that we argue should inform planning, implementing, and embedding complex interventions in healthcare settings. In common with other researchers [2,19,57,58] we argue that potential barriers to and facilitators for introduction, implementation, and embedding of complex interventions should be considered *a priori* when developing the implementation strategy, ideally by researchers in partnership with service providers [57]. Implementation theory provides comprehensive advice on factors to focus on in order to maximize the likely effectiveness of implementation [2,9,58]. Barriers and facilitators examined should expressly include contextual factors, including local and national policy directives and planned responses to these during the anticipated implementation period. Introducing complex interventions that rely on active service user participation will require explicit consideration of what partnership models mean, including how such models are integrated into professional practice and whether this is a necessary condition to secure the intended intervention outcomes. Settings where there are persistent staff shortages, where teams are in conflict or where new teams are forming are likely to experience difficulty with each of the generative mechanisms identified by NPT [2,11]. These represent clear barriers to implementation and so require focused and practical solutions in advance of planned program change [57]. Preparing staff for program change through cascade training can be effective, but all groups involved need to be supported to ensure participation. Refresher training should be scheduled to facilitate reviews of progress and problem solving where necessary, and ensure that new staff are trained in the intervention and its implementation [9,58]. Where implementation of interventions is dependent on multiple providers, time needs to be invested in reaching agreement on who will take responsibility for delivery of specific components and determining how implementation and its effectiveness will be monitored. Finally, as noted at the outset, developing and evaluating complex interventions requires attention not only on the theory and practice of the intervention or on the 'fidelity’ problem, explicit consideration also needs to be given to the implementation process so as to effect change at organizational, practice, and service delivery levels. Combining theory driven approaches to implementation and their refinement through empirical examination of what happens in concrete instances of actual interventions represents a way forward [57,58].

## Competing interests

The authors declare that they have no competing interests.

## Authors' contributions

AF, CM, JD, DJC, MG, and AJ F designed the process evaluation study. DJC, RH, ES, and GH collected and analysed the data in the study. DJC and MG developed the manuscript. AF, CM, RH, ES, JD, GH, and AJF reviewed the manuscript and made recommendations for changes and additions. All authors read and approved the final manuscript.
